# Risk Factors Associated with Traumatic Brain Injury and Implementation of Guidelines for Requesting Computed Tomography After Head Trauma Among Children in France

**DOI:** 10.1001/jamanetworkopen.2023.11092

**Published:** 2023-05-02

**Authors:** Stanislas Roche, Amandine Crombé, Axel Benhamed, Jean-François Hak, Alexia Dabadie, Clémence Fauconnier-Fatus, Adelaïde Rega, Grégoire Pech-Gourg, Karim Tazarourte, Mylène Seux, Adrien Acquier, Guillaume Gorincour

**Affiliations:** 1 IMADIS, Lyon, Bordeaux, Marseille, Dijon, France; 2 Department of Radiology, Pellegrin University Hospital, Bordeaux, France; 3 Models in Oncology (MONC) Team, INRIA Bordeaux Sud-Ouest, CNRS UMR 5251 & Bordeaux University, Talence, France; 4 Service SAMU-Urgences, Centre Hospitalier Universitaire Édouard Herriot, Hospices Civils de Lyon, Lyon, France; 5 Assistance Publique—Hôpitaux de Marseille, Marseille, France; 6 LIIE, Aix Marseille University, Marseille, France; 7 CERIMED, Aix Marseille University, Marseille, France; 8 CHU de Dijon, Dijon, France; 9 ELSAN, Clinique Bouchard, Marseille, France

## Abstract

**Importance:**

Pediatric traumatic brain injuries (TBIs) are a leading cause of death and disability. The Pediatric Emergency Care Applied Research Network (PECARN) guidelines provide a framework for requesting head computed tomography (HCT) after pediatric head trauma (PHT); however, quantitative data are lacking regarding both TBIs found on HCT and justification of the HCT request according to the PECARN guidelines.

**Objectives:**

To evaluate the types, frequencies, and risk factors for TBIs on HCT in children referred to emergency departments (EDs) who underwent HCT for PHT and to evaluate quality of HCT request.

**Design, Setting, and Participants:**

This multicenter, retrospective cohort study included patients younger than 18 years who underwent HCT for PHT who were referred to 91 EDs during on-call hours between January 1, 2020, to May 31, 2022. Data were analyzed between July and August 2022.

**Exposure:**

All radiological reports with pathologic findings were reviewed by 4 senior radiologists. Six hundred HCT requests filled by emergency physicians were randomly sampled to review the examination justification according to the PECARN guidelines.

**Main Outcomes and Measures:**

Associations between TBIs, age, sex, and Glasgow Coma Scale (GCS) were investigated using univariable χ^2^ and Cochrane-Armitage tests. Multivariable stepwise binary logistic regressions were used to estimate the odds ratio (ORs) for intracranial hemorrhages (ICH), any type of fracture, facial bone fracture, and skull vault fracture.

**Results:**

Overall, 5146 children with HCT for PHT were included (median [IQR] age, 11.2 [4.7-15.7] years; 3245 of 5146 [63.1%] boys). ICHs were diagnosed in 306 of 5146 patients (5.9%) and fractures in 674 of 5146 patients (13.1%). The following variables were associated with ICH in multivariable analysis: GCS score of 8 or less (OR, 5.83; 95% CI, 1.97-14.60; *P* < .001), extracranial hematoma (OR, 2.54; 95% CI, 1.59-4.02; *P* < .001), skull base fracture (OR, 9.32; 95% CI, 5.03-16.97; *P* < .001), upper cervical fracture (OR, 19.21; 95% CI, 1.79-143.59; *P* = .006), and skull vault fracture (OR, 35.64; 95% CI, 24.04-53.83; *P* < .001). When neither extracranial hematoma nor fracture was found on HCT, the OR for presenting ICH was 0.034 (95% CI, 0.026-0.045; *P* < .001). Skull vault fractures were more frequently encountered in children younger than 2 years (multivariable OR, 6.31; 95% CI, 4.16-9.66; *P* < .001; reference: children ≥12 years), whereas facial bone fractures were more frequently encountered in boys older than 12 years (multivariable OR, 26.60; 95% CI, 9.72-109.96; *P* < .001; reference: children younger than 2 years). The justification for performing HCT did not follow the PECARN guidelines for 396 of 589 evaluable children (67.2%) for requests filled by emergency physicians.

**Conclusion and Relevance:**

In this cohort study of 5146 children who underwent HCT for PHT, knowing the odds of clinical and radiological features for ICHs and fractures could help emergency physicians and radiologists improve their image analysis and avoid missing significant injuries. The PECARN rules were not implemented in nearly two-thirds of patients.

## Key Points

**Question:**

What are the findings on computed tomography for pediatric head trauma and their risk factors?

**Finding:**

This cohort study included 5146 children who underwent head computed tomography for traumatic brain injury, and 5.9% showed intracranial hemorrhage (ICH), with the following statistically significant associations—Glasgow score of 8 or less, extracranial hematoma, skull base fracture, upper cervical fracture, and skull vault fracture. When neither extracranial hematoma nor fracture was found, the risk for presenting ICH was divided by nearly 30.

**Meaning:**

These findings suggest that knowing the odds of clinical and radiological features for ICHs and fractures could help emergency physicians and radiologists improve their image analysis and avoid missing significant injuries.

## Teaser

This cohort study examines the types, frequencies, and risk factors for traumatic brain injuries (TBIs) on head computed tomography (CT) in children referred to emergency departments.

## Introduction

Traumatic brain injury (TBI) is the most common childhood injury and the leading cause of death in children older than 1 year in developed countries.^1,2^ In the United States, TBIs are responsible for approximately 1500 deaths and more than 640 000 emergency department (ED) visits annually in children younger than 15 years of age.^3^ Most (95%) pediatric head trauma (PHT) cases are mild (Glasgow coma scale [GCS], 13-15), but less than 10% demonstrate an intracranial lesion (including nearly 1.5% of intracranial hemorrhage [ICH]), of which 16% require neurosurgical intervention.^4,5,6^

Due to its accessibility, acquisition speed, and diagnostic performance, head computed tomography (HCT) is the first imaging modality for PHT.^7^ In France, HCT requests are guided by the 2014 National Institute of Health and Care Excellence (NICE)^8^ and the Pediatric Emergency Care Applied Research Network (PECARN) guidelines^4^ to limit radiation exposure and human, material, and organizational associated costs.^9,10^ In parallel, the increasing number of ED visits and access to scanners has considerably increased the use of CT in recent decades, especially in children.^11^ Consequently, radiation exposure increased, leading to higher oncological risk.^12,13^

The recent raise of teleradiology has reorganized imaging accessibility in the ED. Teleradiology uses interoperable information and technology tools that enable rapid collection of large multicentric data sets, especially when structured requests and reports are made.^14^ Since the publication of the PECARN guidelines in 2009,^4^ clinical investigations have emphasized their sensitivity, safety, and validity,^15,16^ with a reduction of HCT rate in patients younger than 2 years without increasing the number of missed clinically important TBIs.^17^ The guidelines have also been acknowledged for their usefulness and ease of use for rapid decision-making by the medical staff,^15,18^ although their specificity may be lower than the specificity from clinical suspicion.^15^ However, data from the radiologic perspective (ie, once HCT has been performed) are lacking. Yet, knowing and quantifying the strength of associations between clinical and radiological features (notably extracranial hemorrhage [ECH], ICH, and fractures on HCT) could be clinically useful for both emergency physicians and radiologists to improve their interpretation and subsequent decision. Furthermore, such multicentric radiologic cohorts could provide an overview of the implementation of the PECARN rules in the HCT request written by emergency physicians in France.

Therefore, taking advantage of our teleradiological structure dedicated to emergency imaging for 91 French EDs, we aimed to review the findings on HCT performed for PHT in children.^14^ Our first objective was to determine the types, frequencies, and risk factors for TBI (ICH and fracture, diagnosed on HCT) in children referred to the ED for PHT and who all underwent HCT. Our secondary objectives were to assess the prevalence and distribution of TBIs by age category, sex, and GCS, and to evaluate the quality of the indication provided by emergency physicians to justify performing HCT in agreement with the PECARN guidelines.^4,8^

## Methods

### Study Design

This study follows the Strengthening the Reporting of Observational Studies in Epidemiology (STROBE) reporting guideline for cohort studies.^19^ The National Radiological Ethics Review Board approved this multicenter cross-sectional retrospective study, and informed consent was waived because data were anonymized. Patients were informed that their data could be anonymized and reused in a study.

We included all consecutive children (younger than 18 years) referred to the ED of 91 partner hospitals of our teleradiology company, who had a PHT between January 1, 2020, and May 31, 2022, during on-call hours and prospectively included in a dedicated head trauma teleradiological workflow, which started on January 1, 2020, and includes the possibility to fill a standardized HCT request form for ED physicians and encodes the main radiological conclusions in a database. As a result, we only included patients for whom a HCT was performed.

HCTs were requested by ED physicians for children suspected of having TBI according to French guidelines or performed following case-by-case discussion and agreement between the ED physician and radiologist.^5^ Exclusion criteria were no available HCT, HCT performed in adults, nontraumatic setting, and follow-up HCT of an already-documented TBI.

### Teleradiological Workflow

Our teleradiology company works with French public and private partner hospitals that transfer us the responsibility to validate, provide protocol, and interpret CT and MR requested in an emergency setting. Consequently, patients for whom no CT or MR is requested did not appear in our database. No funding was necessary or searched for because examinations are performed within current patient care.

The interpretation protocol met the French recommendations for teleradiology.^20^ Standardized requests with clinical and biological information were received via ITIS software (Deeplink Medical). Each request was checked by an on-call radiologist. Examinations were then performed on-site, and images were securely transferred via a private server to a local picture archiving and communication system (Carestream Health 11.0). HCTs were performed using 16-slice or 64-slice multidetector CT scanners with standardized protocols adapted to children’s morphometry (eTable 1 in Supplement 1). HCTs were then interpreted in a dedicated interpretation center (Lyon, Bordeaux, Marseille, Rennes, and Saint-Etienne, France) by 1 of the 15 radiologists working during the on-call periods (ie, during night-time [from 6 PM to 8 AM], except during the weekend or French bank holidays where the on-call periods last 24 hours). All on-call radiologists have at least 2.5 years of experience in emergency radiology and 6 months of residency in pediatric radiology. If in doubt, they could seek a second opinion from a senior pediatric radiologist during the on-call period.

### Data Collection

The following information was retrieved from the database: patient age (in years, categorized as <2, ≥2 and <6, ≥6 and <12, and ≥12),^21^ sex, GCS, emergency level of the HCT request (categorized as extreme, usual, and organizational [ie, that can be delayed compared with other emergencies, but need to be performed to decide whether the patients can be discharged]), need for contrast media, and primary conclusion (categorized as normal, abnormal unrelated to trauma, abnormal related to trauma). The form for HCT request is available in eAppendix in Supplement 1.

We filtered the radiological reports labeled as abnormal related to trauma in the database and extracted their results and conclusion. Four senior radiologists with expertise in pediatric radiology (A.A. and S.R., 1 year; A.D., 7 years; and G.G., 15 years) reviewed the database and the radiological reports (including the Results and Conclusion sections in a free text format) to encode the abnormal findings related to trauma described by the on-call radiologists as follows: presence of any ICH, extradural hematoma (EDH), subdural hematoma (SDH), subarachnoid hemorrhage (SAH), petechiae, intraparenchymal hematoma (IPH), and intraventricular hemorrhage (IVH), as well as their location and the number of distinct types of ICH. They reported the presence of fractures, classified as facial bones, skull vault, skull base, suture disjunction or upper cervical spine (UCS), their location, association with ICH (same or opposite size), and the number of distinct fracture types. Radiologists reported the presence of pneumocephalus, subfalcine, temporal and central herniations, cerebral edema, hydrocephalus, and diffuse axonal lesions (DAL). All the aforementioned fractures and intra-cranial lesions defined TBIs in this study. Lastly, they reported extracranial hematoma (ECH; ie, cephalhematoma and soft tissue swelling). The 4 senior pediatric radiologists had full access to the HCT images to verify the findings described in the reports in case of ambiguities.

Moreover, 600 patients were randomly selected (150 patients per senior pediatric radiologist), and the indication sections from the standardized request filled by emergency physicians were retrospectively reviewed to collect information required to reproduce the clinical algorithms for HCT request in children recommended in France, which are directly derived from the PECARN decision rules and include age; GCS of 14 or less; clinical signs for basal skull fracture or depressed skull fracture or nonfrontal scalp hematoma; loss of consciousness; vomiting; severe trauma mechanism; significant cephalalgia; abnormal behavior according to parents; and clinical alterations (eFigure 1 in Supplement 1).^4,5,8^ After retrospectively applying the algorithms, they reported their conclusions categorized as recommended HCT, in-hospital follow-up, or discharge. In addition, they reviewed the radiological reports of this subsample of the study population in order to encode the main pathological findings related to trauma as described by the on-call emergency radiologist during practice. Again, if needed, ambiguities in the radiological report were solved by careful reviews of the initial imaging by the senior pediatric radiologists.

### Statistical Analysis

In the whole cohort, associations between TBIs on HCT and categorical variables were performed with the χ^2^ test (complemented with Cochrane-Armitage tests for testing associations between binary and ordinal variables). Adjustment for multiple comparisons was achieved for the univariable analyses with the Benjamini-Hochberg procedure to control the risk of false discovery with a level α = .05. Odds ratios (ORs) with 95% CI were estimated using univariable binary logistic regression for the following outcomes: any type of fracture, facial bone fracture, skull vault fracture, and ICH. We chose to split TBIs into those different outcomes because radiologists are used to analyzing HCT on bone kernel and brain parenchyma kernel separately and to provide insight into the specific odds for fractures and ICH. We hypothesized that the children’s behavior was influenced by age and sex, the odds for fracture was increased when a ECH was present, and the odds for an ICH was increased when an ECH or a fracture was present. Consequently, the following variables were investigated: (1) age, sex, GCS, and ECH for any type of fracture and the main 2 subtypes of fracture; and (2) age, sex, GCS, ECH, any type of fracture, number of distinct fracture types, and all the subtypes of fracture for ICH. Additionally, we created a composite variable from the ECH and any type of fracture variables to estimate the univariable OR for ICH when no ECH and no fracture were seen on HCT.

Afterwards, stepwise backward-forward procedures minimizing the Akaike information criterion were applied to estimate multivariable ORs for variables significantly associated with ICH and fracture in the univariable assessment using the MASS package version 7.3-54 (R Project for Statistical Computing). Lastly, in the random sample of 600 patients whose HCT requests were reviewed, the number and proportion of patients with each type of conclusion were assessed. In order to verify the representativeness of the pathological findings in the sample compared with the entire cohort, we compared the distribution of ICH and fracture in the 2 populations with χ^2^ tests. Patients with missing data regarding the potential factors associated with TBI or the outcomes were removed from the corresponding statistical testing. Statistical analyses were performed with R version 4.1.2 (R Project for Statistical Computing). All tests were 2-tailed, and *P* < .05 was deemed significant. Data were analyzed between July and August 2022.

## Results

### Study Population

The head trauma workflow comprised 110 938 patients. After excluding 103 445 adults and 2347 patients imaged before January 1, 2020, 5146 patients were included (median [IQR] age, 11.2 [4.7-15.7] years; 3245 of 5146 boys [63.1%]) (Table 1). Of them, 860 of 5146 patients (16.7%) demonstrated abnormal findings related to trauma on HCT, and 600 (11.7%) patients were randomly sampled to investigate the agreement between the HCT requests filled by emergency physicians and the PECARN rules, respectively (Figure). The head was the only scanned area in 5130 patients (99.7%), and no contrast media was used in 5079 patients (98.7%).

**Table 1.** Characteristics of the Entire Cohort

Characteristics	Patients, No. (%) (N = 5146)
**Clinical characteristics**	
Age, median (IQR), y	11.2 (4.7-15.7)
Age categories, y	
0-1	659 (12.8)
2-5	927 (18)
6-11	1137 (22.1)
12-17	2423 (47.1)
Sex (n = 5144)^a^	
Male	3245 (63.1)
Female	1899 (36.9)
Glasgow Coma Scale (n = 3643)^b^	
15	3211 (88.1)
13-14	301 (8.3)
9-12	91 (2.5)
3-8	40 (1.1)
**Examination-related characteristics**	
Emergency level of the HCT request	
Extreme emergency (level 1)	134 (2.6)
Usual emergency (level 2)	4727 (91.9)
Organizational emergency (level 3)	285 (5.5)
Contrast medium injection, yes	67 (1.3)
Other acquisition(s) performed during CT examination^c^	
Abdomen pelvic	1 (<0.1)
Thorax abdomen pelvic	11 (0.2)
Chest	4 (0.1)

Abbreviations: CT, computed tomography; HCT, head computed tomography.

^a^ Patients’ sexes were unknown for 2 patients in the full cohort.

^b^ Glasgow Coma Scales were missing for 1503 patients in the full cohort.

^c^ Head computed tomography was systematically performed in all patients included in the study.

**Figure.**
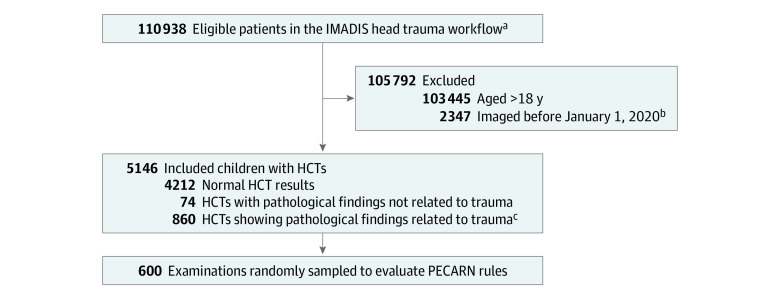
Study Flowchart Abbreviations: HCT, head computed tomography scan; PECARN, Pediatric Emergency Care Applied Research Network. ^a^The head trauma workflow includes a standardized HCT form for the emergency physicians and encodes the main radiological conclusions in a database. ^b^Examinations before January 1, 2020, were removed because the head trauma workflow was not deployed in its current version before this date. ^c^Imaging was available to readers if a verification of the report was needed.

### Depiction of TBIs Found on HCT

ICH was found in 306 of 5146 patients (5.9%) and mostly consisted of SDH (129 [2.5%]), EDH (126 [2.4%]) and SAH (82 [1.6%]) (Table 2). At least 2 different ICH types were found in 94 patients (1.8%) (mainly EDH and SDH).

**Table 2.** Radiological Findings in the Entire Cohort

Characteristics	Patients, No. (%) (N = 5146)
Pathological findings	
No	4212 (81.8)
Yes, related to the trauma	860 (16.7)
Yes, but unrelated to trauma	74 (1.4)
ICH	306 (5.9)
ICH types	
Subdural hematoma	129 (2.5)
Extradural hematoma	126 (2.4)
Subarachnoid hemorrhage	82 (1.6)
Petechiae	66 (1.3)
Intraparenchymal hemorrhage	19 (0.4)
Intraventricular hemorrhage	5 (0.1)
No. of distinct ICH types	
1	212 (4.1)
2	68 (1.3)
3	25 (0.5)
4	1 (<0.1)
Fracture	674 (13.1)
Fracture types	
Facial bone	329 (6.4)
Skull vault	322 (6.3)
Skull base	126 (2.4)
Suture disjunction	25 (0.5)
Upper cervical spine	6 (0.1)
No. of distinct fracture types	
1	555 (10.8)
2	106 (2.1)
3	11 (0.2)
4	2 (<0.1)
Other intracranial traumatic lesions	
Pneumocephalus	85 (1.7)
Subfalcine herniation	15 (0.3)
Temporal herniation	7 (0.1)
Central herniation	3 (0.1)
Cerebral edema	11 (0.2)
Hydrocephalus	5 (0.1)
Diffuse axonal lesions	4 (0.1)
Extracranial hematoma	270 (5.2)

Abbreviation: ICH, intracranial hemorrhage.

A fracture was found in 674 of 5146 patients (13.1%) and mostly involved facial bones (329 [6.4%]), skull vault (322 [6.3%]), and skull base (126 [2.4%]). At least 2 different fracture types were found in 119 patients (2.3%) (mainly skull base and vault). Skull vault fractures were mostly simple and linear (251 of 322 [78%]), with a depressed skull fracture in 48 of 322 patients (14.9%). The same-side and opposite-side ICHs were reported in 158 of 322 (49.1%) and 23 of 322 (7.1%) patients with skull vault fractures, respectively. The most frequent nonhemorrhagic intracranial lesions were pneumocephalus (85 of 5146 [1.7%]), subfalcine herniation (15 of 5146 [0.3%]), and cerebral edema (12 of 5126 [0.2%]). An ECH was found in 279 of 5146 patients (5.4%).

### Association of Clinical Variables With TBIs

#### Age

The age groups were significantly associated with ICH with a downward trend, particularly EDH, SDH, and SAH. Children younger than 2 years showed the highest rates of ICH (84 of 659 [12.7%] vs 88 of 2423 [3.6%] older than 12 years; *P* < .001), EDH (37 of 659 [5.6%] vs 33 of 2423 [1.4%] older than 12 years; *P* < .001), SDH (40 of 659 [6.1%] vs 40 of 2423 [1.7%] older than 12 years; *P* < .001) and SAH (18 of 659 [2.7%] vs 26 of 2423 [1.1%] older than 12 years; *P* = .007). Skull vault fractures and ECH were significantly more frequent at younger ages, with the highest prevalence in children younger than 2 years (116 of 659 [17.6%] younger than 2 years vs 62 of 2423 [2.6%] older than 12 years; *P* < .001; and 82 of 659 [12.4%] younger than 2 years vs 81 of 2423 [3.3%] older than 12 years; *P* < .001; respectively). Conversely, facial bone fractures were associated with age but with an upward trend (217 of 2423 [9%] older than 12 years vs 4 of 659 [0.6%] younger than 2 years; *P* < .001) (eTable 2 in Supplement 1).

#### Sex

No association was found between sex and any ICH. However, boys displayed significantly more fractures than girls (479 of 3245 [14.8%] vs 195 of 1899 [10.3%]; *P* < .001), especially more facial bone fractures (242 of 3245 [7.5%] vs 87 of 1899 [4.6%]; *P* < .001), skull base fractures (94 of 3245 [2.9%] vs 32 of 1899 [1.7%]; *P* = .04) and skull vault fractures (225 of 3245 [6.9%] vs 97 of 1899 [5.1%]; *P* = .04), as well as more ECH (189 of 3245 [5.8%] vs 81 of 1899 [4.3%]; *P* = .048) (eTable 3 in Supplement 1).

#### GCS

GCS scores were available for 3643 of 5146 patients (70.8%). In patients with a GCS of 15, ICH was encountered in 184 of 3211 patients (5.7%), including 74 of 3211 (2.3%) EDHs and 80 of 3211 (2.5%) SDHs. In patients with a GCS of 13 to 14, ICH was encountered in 28 of 301 (9.3%), including 12 of 301 (4.0%) EDHs and 17 of 301 (5.6%) SDHs. In patients with a GCS of 9 to 12, ICH was encountered in 11 of 91 (12.1%), including 4 of 91 (4.4%) EDHs and 5 of 91 (5.5%) SDHs, and in patients with a GCS of 3 to 8, ICH was encountered in 7 of 40 (17.5%), including 2 of 40 (5.0%) EDHs and 2 of 40 (5.0%) SDHs. GCS scores were significantly associated with ICH, EDH, SDH, SAH, and petechiae with a downward trend (*P* < .001, *P* = .05, *P* = .006, *P* < .001, and *P* < .001, respectively). The incidences of skull vault and skull base fractures slightly increased as GCS score decreased, with 190 of 3211 (5.9%) skull vault fractures and 66 of 3211 (2.1%) skull base fractures in patients with a GCS of 15 compared 40 of 432 (9.3%) skull vault fractures and 20 of 432 (4.6%) skull base fractures in patients with a GCS less than 15 (skull vault fracture: *P* = .02; skull base fracture: *P* = .05) (eTable 4 in the Supplement).

### Multivariable Associations Between Patterns of Injuries and Clinical Features

#### Fractures

In the multivariable analysis (Table 3), the following variables were associated with any fracture: boys (OR, 1.48 [95% CI, 1.17-1.88]; *P* = .001), GCS of 13 to 14 (OR, 1.83 [95% CI, 1.3-2.53] *P* < .001; reference, GCS = 15) and ECH (OR, 26.60 [95% CI, 19.06-37.68]; *P* < .001). Facial bone fractures were associated with boys (OR, 1.49 [95% CI, 1.09-2.06]; *P* = .013), age with gradual increase in ORs compared with the 0 to 1 year group (2-5 years: OR, 11.35 [95% CI, 3.94-48.04]; *P* < .001; 6-11 years: OR, 14.16 [95% CI, 5.03-59.38]; *P* < .001; 12-17 years: OR, 26.60 [95% CI, 9.72-109.96]; *P* < .001), and ECH (OR, 9.97 [95% CI, 6.75-14.64]; *P* < .001).

**Table 3.** Univariable and Multivariable Associations With Any Fracture and Main Subtypes of Fracture on Head Computed Tomography

Characteristics	Patient, No. (%)		Univariable analysis			Multivariable analysis	
	Absent	Present	OR (95%CI)	*P* value	Adjusted *P* value^a^	OR (95%CI)	*P* value
Any type of fracture, total No	4472	674	NA	NA		NA	NA
Age, y							
12-17	2140 (47.9)	283 (42.0)	1 [Reference]	NA	<.001	Removed^b^	Removed^b^
0-1	529 (11.8)	130 (19.3)	1.86 (1.48-2.33)	<.001			
2-5	1002 (22.4)	135 (20.0)	1.02 (0.82-1.26)	.87			
6-11	801 (17.9)	126 (18.7)	1.19 (0.95-1.49)	.13			
Sex^c^							
Girls	1704 (38.1)	195 (28.9)	1 [Reference]	NA	<.001	1 [Reference]	NA
Boys	2766 (61.9)	479 (71.1)	1.51 (1.27-1.81)	<.001		1.48 (1.17-1.88)	.001
Glasgow coma scale^d^							
15	2806 (88.9)	405 (83.5)	1 [Reference]	NA	.001	1 [Reference]	NA
13-14	239 (7.6)	62 (12.8)	1.80 (1.32-2.41)	<.001		1.83 (1.3-2.53)	<.001
9-12	77 (2.4)	14 (2.9)	1.26 (0.68-2.18)	.43		1.36 (0.69-2.46)	.34
3-8	36 (1.1)	4 (0.8)	0.77 (0.23-1.94)	.62		0.71 (0.19-2.00)	.57
Extracranial hematoma							
No	4410 (98.6)	466 (69.1)	1 [Reference]	NA	<.001	1 [Reference]	NA
Yes	62 (1.4)	208 (30.9)	31.75 (23.68-43.13)	<.001		26.60 (19.06-37.68)	<.001
Facial bone fracture, total No.	4817	329					
Age, y							
0-1	655 (13.6)	4 (1.2)	1 [Reference]	NA	<.001	1 [Reference]	NA
2-5	1071 (22.2)	66 (20.1)	10.09 (4.15-33.3)	<.001		11.35 (3.94-48.04)	.004
6-11	885 (18.4)	42 (12.8)	7.77 (3.12-25.94)	<.001		14.16 (5.03-59.38)	<.001
12-17	2206 (45.8)	217 (66.0)	16.11 (6.82-52.37)	<.001		26.60 (9.72-109.96)	<.001
Sex^c^							
Girls	1812 (37.6)	87 (26.4)	1 [Reference]	NA	<.001	1 [Reference]	NA
Boys	3003 (62.4)	242 (73.6)	1.68 (1.31-2.17)	<.001		1.49 (1.09-2.06)	.01
Glasgow coma scale^d^							
15	3017 (88.4)	194 (83.6)	1 [Reference]	NA	.03	Removed^b^	Removed^b^
13-14	270 (7.9)	31 (13.4)	1.79 (1.18-2.62)	.004			
9-12	86 (2.5)	5 (2.2)	0.90 (0.32-2.04)	.83			
3-8	38 (1.1)	2 (0.9)	0.82 (0.13-2.70)	.78			
Extracranial hematoma							
No	4626 (96)	250 (76.0)	1 [Reference]	NA	<.001	1 [Reference]	NA
Yes	191 (4)	79 (24.0)	7.65 (5.70-10.21)	<.001		9.97 (6.75-14.64)	<.001
Skull vault fracture, total No	4824	322					
Age, y							
12-17	2361 (48.9)	62 (19.3)	1 [Reference]	NA	<.001	1 [Reference]	NA
0-1	543 (11.3)	116 (36.0)	8.14 (5.92-11.28)	<.001		6.31 (4.16-9.66)	<.001
2-5	1076 (22.3)	61 (18.9)	2.16 (1.50-3.10)	<.001		3.24 (2.11-4.99)	<.001
6-11	844 (17.5)	83 (25.8)	3.74 (2.68-5.27)	<.001		2.11 (1.36-3.28)	<.001
Sex^c^							
Girls	1802 (37.4)	97 (30.1)	1 [Reference]	NA	.01	1 [Reference]	NA
Boys	3020 (62.6)	225 (69.9)	1.38 (1.09-1.78)	.009		1.59 (1.14-2.23)	.007
Glasgow coma scale^d^							
15	3021 (88.5)	190 (82.6)	1 [Reference]	NA	.06	Removed^b^	Removed^b^
13-14	273 (8)	28 (12.2)	1.63 (1.06-2.43)	.02			
9-12	82 (2.4)	9 (3.9)	1.75 (0.81-3.35)	.12			
3-8	37 (1.1)	3 (1.3)	1.29 (0.31-3.61)	.67			
Extracranial hematoma							
No	4685 (97.1)	191 (59.3)	1 [Reference]	NA	<.001	1 [Reference]	1 [Reference]
Yes	139 (2.9)	131 (40.7)	23.12 (17.50-30.59)	<.001		17.97 (12.76-25.34)	<.001

Abbreviations: GCS, Glasgow coma scale; OR, odds ratio.

^a^ Univariable *P* values were adjusted for multiple comparisons (herein 4 tests for each outcome) and corresponded to the overall χ^2^ *P* value.

^b^ Those variables removed from the multivariable stepwise analysis during a step.

^c^ The sex of 2 children were unknown (both without any fracture), and those observations were removed from the analysis.

^d^ A total of 1503 GCSs were missing, which were distributed as follows: 1314 of 1503 (87.4%) without any fracture and 189 of 1503 (12.6%) with at least 1 fracture; 1404 of 1503 (93.4%) without skull base fracture and 97 of 1503 (6.4%) with skull base fracture; 1411 of 1503 (93.9%) without skull vault fracture and 92 of 1503 (6.1%) with skull vault fracture; those observations were removed from the corresponding tests.

In addition, skull vault fractures were associated with boys (OR, 1.59 [95% CI, 1.14-2.23]; *P* = .007) and ECH (OR, 17.97 [95% CI, 12.76-25.34]; *P* < .001). They were also associated with age as well as with facial bone fractures, as there was a gradual increase in OR with younger age (6 to 11 years: OR, 2.11 [95% CI, 1.36-3.28]; *P* < .001; 2 to 5 years: OR, 3.24 [95% CI, 2.11-4.99]; *P* < .001; 0 to 1 year: OR, 6.31 [95% CI, 4.16-9.66]; *P* < .001; reference: 12-17 years).

#### ICH

Overall, when neither ECH nor fracture was found, the OR for presenting any ICH was 0.034 (95% CI, 0.026-0.045; *P* < .0001). In the multivariable analysis, the following variables remained associated with any ICH: GCS score of 8 or less (OR, 5.83 [95% CI, 1.97-14.6]; *P* < .001); ECH (OR, 2.54 [95% CI, 1.59-4.02]; *P* < .001); skull base fracture (OR, 9.32 [95% CI, 5.03-16.97]; *P* < .001); UCS fracture (OR, 19.2 [95% CI, 1.79-143.59]; *P* = .006); and skull vault fracture (OR, 35.64 [95% CI, 24.04-53.83]; *P* < .001) (Table 4). eFigures 2 and 3 in Supplement 1 show HCTs with typical suites of TBIs.

**Table 4.** Univariable and Multivariable Associations With Intracranial Hemorrhage (ICH) on Head Computed Tomography

Characteristics	Patients, No. (%)		Univariable OR (95% CI)	*P* value	Adjusted *P* value^a^	Multivariable OR (95% CI)	*P* value
	Patients without ICH (n = 4840)	Patients with ICH (n = 306)					
Age, y							
12-17	2335 (48.2)	88 (28.8)	1 [Reference]	NA	<.001	1 [Reference]	[Reference]
0-1	575 (11.9)	84 (27.5)	3.88 (2.83-5.3)	<.001		1.35 (0.82-2.2)	.23
2-5	866 (17.9)	61 (19.9)	1.87 (1.33-2.61)	<.001		0.73 (0.43-1.21)	.23
6-11	1064 (22)	73 (23.9)	1.82 (1.32-2.5)	<.001		1.51 (0.98-2.34)	.06
Sex^b^							
Female	1798 (37.2)	101 (33)	1 [Reference]	NA	0.16	NA	NA
Male	3040 (62.8)	205 (67)	1.2 (0.94-1.54)	0.14		NA	NA
GCS^c^							
15	3027 (88.7)	184 (80)	1 [Reference]	NA	<.001	1 [Reference]	[Reference]
13-14	273 (8)	28 (12.2)	1.69 (1.09-2.52)	.01		1.48 (0.84-2.49)	.16
9-12	80 (2.3)	11 (4.8)	2.26 (1.12-4.15)	.01		2.31 (0.91-5.25)	.06
3-8	33 (1)	7 (3)	3.49 (1.4-7.54)	.003		5.83 (1.97-14.6)	<.001
Extracranial hematoma							
No	4667 (96.4)	209 (68.3)	1 [Reference]	NA	<.001	1 [Reference]	[Reference]
Yes	173 (3.6)	97 (31.7)	12.52 (9.4-16.62)	<.001		2.54 (1.59-4.02)	<.001
Any fracture^d^							
No	4385 (90.6)	87 (28.4)	1 [Reference]	NA	<.001	NA	NA-
Yes	455 (9.4)	219 (71.6)	24.26 (18.65-31.8)	<.001		NA	NA
No. of distinct fracture types^d^							
0	4385 (90.6)	87 (28.4)	1 [Reference]	NA	<.001	NA	NA
1	402 (8.3)	153 (50)	19.18 (14.5-25.52)	<.001		NA	NA
≥ 2	53 (1.1)	66/306 (21.6)	62.77 (41.39-95.86)	<.001		NA	NA
Facial bone fracture							
No	4548 (94)	269 (87.9)	1 [Reference]	NA	<.001	Removed^e^	Removed^e^
Yes	292 (6)	37 (12.1)	2.14 (1.47-3.04)	<.001		NA	NA
Skull base fracture							
No	4772 (98.6)	248 (81)	1 [Reference]	NA	<.001	1 [Reference]	[Reference]
Yes	68 (1.4)	58 (19)	16.41 (11.28-23.82)	<.001		9.32 (5.03-16.97)	<.001
UCS fracture							
No	4837 (99.9)	303 (99)	1 [Reference]	NA	<.001	1 [Reference]	[Reference]
Yes	3 (0.1)	3 (1)	15.96 (2.94-86.57)	<.001		19.21 (1.79-143.59)	.006
Skull vault fracture							
No	4702 (97.1)	122 (39.9)	1 [Reference]	NA	<.001	1 [Reference]	[Reference]
Yes	138 (2.9)	184 (60.1)	51.39 (38.76-68.51)	<.001		35.64 (24.04-53.38)	<.001
Suture disjunction							
No	4828 (99.8)	293 (95.8)	1 [Reference]	NA	<.001	Removed^e^	Removed^e^
Yes	12 (0.2)	13 (4.2)	17.85 (8.03-40.02)	<.001		NA	NA

Abbreviations: GCS, Glasgow coma scale; ICH, intracranial hemorrhage; NA, not applicable; OR, odds ratio; UCS, upper cervical spine.

^a^ Those *P* values in italic were adjusted for multiple comparisons (herein 11 tests) and corresponded to the overall χ^2^ *P* value.

^b^ The sex of 2 children was unknown (both in the no ICH group) and those observations with missing data were removed from the analysis.

^c^ 1503 GCSs were missing (namely 1427 of 1503 [9.5%] in the no ICH group and 76 of 1503 [5.1%] in the ICH group), and those observations with missing data were removed from the analysis.

^d^ Any fracture and No. of distinct fracture types were not included in the multivariable analysis because they were strongly redundant and collinear with the other variables related to fracture from which they were created.

^e^ Facial bone fracture and suture disjunction was removed from the multivariable stepwise analysis during a step.

### Analysis of the HCT request

The clinical data collected in the sample of 600 children enabled us to retrospectively reproduce the decision algorithms based on the PECARN rules in 589 of 600 patients (98.2%) whose characteristics are given in eTable 5 in Supplement 1 (11 patients with missing or incomprehensible clinical information). There were no differences in the distributions of ICH and fracture (of any type) between the entire cohort and the sample. According to the indications provided in the requests and the PECARN rules, HCT would have been indicated in 193 of 589 patients (32.8%). In-hospital clinical monitoring (but no HCT) would have been indicated in 170 of 589 patients (28.9%). Finally, discharge with neither HCT nor follow-up would have been theoretically indicated in 226 of 589 patients (38.4%), although 11 of 226 patients (4.9%) displayed ICH and 29 of 226 patients (11.2%) had fractures. The radiological findings in those 3 groups are provided in eTable 6 in Supplement 1.

## Discussion

In this multicenter cohort of children admitted to the ED who underwent HCT for PHT, abnormal findings were reported in 16.7% of patients, ICH in 5.9%, and fractures in 13.1%. Although TBI in children is rare, with interoperable information systems like our teleradiological network, our study is the largest to date to provide macroscopic data in the clinical setting from several EDs about prevalence and types of as well as factors associated with TBI in a population of children who underwent HCT, enabling us to achieve multivariable assessments and to review the quality of the HCT requests in light of the PECARN rules.

Our results are consistent with a large systematic review of patients with mild TBI^3^ where intracranial injury was found in 7.5% of patients. However, skull fractures without any intracranial injury were found in 18.2% of patients vs 9.2% in our study. This could be related to our large proportion of children older than 12 years, who mainly presented with facial trauma. Indeed, compared with the original study,^4^ our population was older (mean [SD] age, 10.1 [5.9] years vs 7.1 [5.5] years), with 12.8% patients younger than 2 years (vs 25%). Herein, the GCS score was 15 for 88.1% of patients vs 97% in the study by Kuppermann et al.^4^ Sex was not studied in this original study, yet boys were overrepresented (63.1%), which is relevant with other studies.^9,22^

Age was inversely associated with the risk of ICH. Indeed, in children younger than 2 years, regardless of sex, the proportions of cranial vault fracture, ECH, EDH, and SDH were the highest. This could be explained by skull anatomy of younger children; the cranium increases 4 times and the face increases 12 times from birth to adulthood.^23^ Consequently, teenagers usually have facial fractures, whereas young children are more likely to have skull fractures.^24,25,26^ Moreover, trauma mechanisms differ across age groups (falls in toddlers vs car crash accidents in teenagers), with a higher prevalence of ICH found with falls.^24^ Herein, the multivariable odds for fracture (of any type) was 1.48 in boys (mostly facial bone fractures), which is consistent with the literature where the predominance of boys is mainly due to their more hazardous physical activity.^27^

Apart from the impact of age and sex, we found that a GCS of 14 or less doubled the odds of ICH, which is consistent with the literature and guidelines.^3^ GCS was inversely proportional to the prevalence of ICH and fractures. However, 184 patients with a GCS of 15 presented with ICH, highlighting the need for additional clinical information and the difficulties for physicians to accurately obtain GCS score in preverbal children.^28^

In our series, the multivariable odds for ICH were 2.54 in the presence of ECH, 9.32 in the presence of cranial base fracture, 19.2 in the presence of UCS fracture, and 35.6 in the presence of cranial vault fracture. Furthermore, presenting an ECH increased the risk of fracture by 32. Moreover, when neither ECH nor fracture was found on HCT, the odds for presenting ICH was divided by nearly 30.

Although these associations are already known,^29^ the values of their ORs highlight their magnitude and could have practical consequences for radiologists, notably higher caution to these warning signs. For instance, Sigmund et al^30^ developed a clinical rule for asymptomatic patients with clinical scalp abnormalities to determine which asymptomatic infants need HCT, and their results are in accordance with ours.

However, detecting ICH on HCT is not systematically clinically relevant since some findings will not lead to special management (eg, small SDHs that self-resolve). Conversely, not detecting an ICH does not automatically imply that a HCT was not valuable since other significant TBI can be detected.

Regarding the analysis of the HCT requests in the subsample of 600 patients, HCT seemed to have performed outside the PECARN guidelines for 396 of children 589 (67.2%) who were evaluable. However, in this outside recommendation group, the prevalence of ICH and any fracture were relatively high (4.8% and 12.1%, respectively), which stresses that those HCT were likely to be indicated. Thus, we hypothesize that performing HCT for those patients was actually required but insufficiently justified in the HCT request by comparison with the current guidelines. Thus, our work emphasizes the need to improve the justification for performing HCT in the request filled by emergency physicians (by better implementing the PECARN rules) as well as the communication time and digital tools (such as more adapted standardized forms for HCT request by teleradiological structures) to find a compromise between the clinical work overload of emergency physicians and the need for radiologists to have the most appropriate and accurate information so that they can achieve the best interpretation. Future studies could investigate the impact of ED crowding and work overload on the correct implementation of the guidelines for requesting HCT.

### Limitations

This study had limitations. First, this was a retrospectively designed study based on prospectively collected radiological requests and reports. Therefore, the GCS was missing for 1503 patients, which may not be random. Additionally, the mechanism of injury and the injury severity score were not prospectively encoded in the standardized form for HCT requests, which prevented us from investigating those covariables in the statistical analyses. Although the 4 senior radiologists had access to images in case of doubtful reports, imaging was not specifically reviewed for the purpose of this work. Consequently, we could not estimate interobserver reliability and ensure that there were no mistakes in the initial reports and that imaging was analyzed according to best practices, eg, by using 3-dimensional volume rendering technique (which significantly increases fracture detection).^31^ Additionally, radiologists who initially interpreted HCT were not all pediatric radiologists; hence, we could not exclude whether some age-related specificities were misinterpreted. ^26,32,33,34^ Although all had at least 2.5 years of experience in emergency radiology and could refer to a senior radiologist for complementary advice during the on-call period.

Second, we investigated abnormal findings and univariable and multivariable associations in a selected population of children requiring HCT according to emergency physicians. Thus, the prevalence of TBIs did not reflect their overall prevalence in the entire population of PHT. Consequently, our data could not directly help develop new decision algorithms on the general population. It could only be applied to a population sharing the same inclusion criteria. Moreover, the multivariable analyses included both clinical and radiological variables assessed on HCT. Therefore, their results could only be applied in patients who already underwent HCT. Third, although HCT is currently the reference standard among imaging techniques for PHT, the sensitivity for identifying DAL (ie, the main cause of posttraumatic neurologic disability) is lower than magnetic resonance imaging.^7,26,35^ Fourth, it must be noted that the outcome used for developing the PECARN rules were clinically important TBIs (ie, leading to death, neurosurgical intervention, intubation for more than 24 hours, and hospital admission for 2 or more days) instead of any TBI as in our study. As our teleradiologic database lacked hospital follow-up, we could not investigate associations with clinically important TBIs.

## Conclusion

In this cohort study of 5166 children who underwent HCT for PHT, 5.9% of patients demonstrated ICH and 13.1% at least 1 fracture. In multivariable analysis, ICH was associated with a GCS of 8 or less, ECH, skull base fracture, upper cervical fracture, and skull vault fracture. According to the information provided by emergency physicians on HCT request, HCT was indicated in 193 of 589 children (32.8%) according to the PECARN rules. Knowing patterns of TBIs and at-risk groups according to the PECARN rules may help emergency physicians better target patients who would benefit from HCT, may help radiologists avoid missing significant injuries, and may improve the justifications for performing HCT and the radiologic interpretations.

## Notes

Supplement 1. **eTable 1.** Standardized Protocols for HCT for Children Across Partner EDs **eAppendix.** Items From the Standardized Form for HCT Request **eFigure 1.** Decision Algorithm **eTable 2.** Associations Between Main Findings on Head Computed Tomography and Patients’ Age **eTable 3.** Associations Between Main Findings on Head Computed Tomography and Patients’ Sex **eTable 4.** Associations Between Main Findings on Head Computed Tomography and Patients’ Glasgow Coma Scale **eFigure 2.** HCT for 21-month-old Patient **eFigure 3.** HCT for 15-year-old Patient **eTable 5.** Characteristics of the 600 HCT Requests Randomly Sampled to Investigate the Implementation of the PECARN Rules **eTable 6.** Patterns of Traumatic Brain Injuries on HCT Diagnosed in the 3 Groups Depending on the Theoretical Management up to PECARN Algorithm as Used in France

Supplement 2.
Data Sharing Statement
