# Supplementary material for: Risk Factors Associated with Traumatic Brain Injury and Implementation of Guidelines for Requesting Computed Tomography After Head Trauma Among Children in France
Source: JAMA Netw Open. 2023 May 2;6(5):e2311092. doi: 10.1001/jamanetworkopen.2023.11092 (PMC10155067; doi:10.1001/jamanetworkopen.2023.11092)
Supplement: Supplement 1. — eTable 1. Standardized Protocols for HCT for Children Across Partner EDs eAppendix. Items From the Standardized Form for HCT Request eFigure 1. Decision Algorithm eTable 2. Associations Between Main Findings on Head Computed Tomography and Patients’ Age eTable 3. Associations Between Main Findings on Head Computed Tomography and Patients’ Sex eTable 4. Associations Between Main Findings on Head Computed Tomography and Patients’ Glasgow Coma Scale eFigure 2. HCT for 21-month-old Patient eFigure 3. HCT for 15-year-old Patient eTable 5. Characteristics of the 600 HCT Requests Randomly Sampled to Investigate the Implementation of the PECARN Rules eTable 6. Patterns of Traumatic Brain Injuries on HCT Diagnosed in the 3 Groups Depending on the Theoretical Management up to PECARN Algorithm as Used in France [file jamanetwopen-e2311092-s001.pdf]

Supplemental Online Content

Roche S, Crombé A, Benhamed A, et al. Risk factors associated with traumatic brain injury and implementation of guidelines for requesting computed tomography after head trauma among children in France. *JAMA Netw Open*. 2023;6(5):e2311092. doi:10.1001/jamanetworkopen.2023.11092

eTable 1. Standardized Protocols for HCT for Children Across Partner EDs
eAppendix. Items From the Standardized Form for HCT Request
eFigure 1. Decision Algorithm
eTable 2. Associations Between Main Findings on Head Computed Tomography and Patients' Age
eTable 3. Associations Between Main Findings on Head Computed Tomography and Patients' Sex
eTable 4. Associations Between Main Findings on Head Computed Tomography and Patients' Glasgow Coma Scale
eFigure 2. HCT for 21-month-old Patient
eFigure 3. HCT for 15-year-old Patient
eTable 5. Characteristics of the 600 HCT Requests Randomly Sampled to Investigate the Implementation of the PECARN Rules
eTable 6. Patterns of Traumatic Brain Injuries on HCT Diagnosed in the 3 Groups Depending on the Theoretical Management up to PECARN Algorithm as Used in France

This supplemental material has been provided by the authors to give readers additional information about their work.

© 2023 Roche S et al. *JAMA Network Open.*

eTable 1. Standardized Protocols for HCT for Children Across Partner EDs

		Age groups				
		0-1 years	1-3 years	3-6 years	6-12 years	>12 years
**Head**	kV (fixed)	120	120	120	120	120
	mAs maximum	130	150	180	240	adult
	Dose modulation	None	None	None	None	none
	iterative and multiplanar reconstruction			Yes		

NOTE.- The head commuted tomography (HCT) protocols were implemented in each center by the IMADIS technical team and systematically used by all radiographers.
Examinations were performed using a non-contrast 1-to-1.25 mm thick acquisition covering the skull, in dorsal decubitus with arms besides the body, was performed and reconstructed to reach 1 mm thickness in bone and brain kernels. Additional CT acquisitions for other traumatized body areas or contrast medium injection were allowed if requested and justified by the emergency department (ED) physicians. In case of request of contrast medium, the contrast agents were Omnipaque 350 (GE Healthcare), Iomeron 400 (Bracco Diagnostics), and Ultravist 370 (Bayer Healthcare). The injected volume was 2 cc/kg for children < 5 kg and 1.5 cc/kg for those ≥ 5 kg.

eAppendix. Items From the Standardized Form for HCT Request

- Age (numeric, in years)
- Sex (categorical: male, female, prefer not to say)
- Date and hour (“yyyy-mm-dd hour:min:sec” UTC format)
- Bleeding disorder (categorical: no, yes: anticoagulation, yes: anti-platelet aggregation, yes: both, yes, other)
- Alcohol intoxication (categorical: yes or no)
- Date and hour of head trauma (free text)
- Eye opening response (numeric: from 1 to 4)
- Best verbal response (numeric: from 1 to 5)
- Best motor response (numeric: from 1 to 6)
- GCS value (numeric: from 3 to 15)
- Suspicion of facial bone fracture (categorical: yes or no)
- If facial bone fracture suspected, where (categorical: orbit, mandible, other)
- Loss of consciousness (categorical: yes or no)
- Focal neurological sign (categorical: yes or no)
- Detail of the focal neurological sign, if present (free text)
- Emergency level (ordinal: extreme, usual, organizational)
- Expected need for contrast medium injection (categorical: yes, no, not sure)
- bHCG dosage (categorical: no, yes-positive, yes-negative, ongoing)
- Available prior imaging (categorical: yes or no)
- Additional information to the indication (free text)

NOTE.- Emergency physicians could validate a request and send it to radiologists without filling all the items.

eFigure 1. Decision Algorithm

Decision algorithms used in French emergency departments for performing head computed tomography (HCT) in children depending on age: (**A**) before 2 years and (**B**) between 2 and 18 years. These algorithms are directly derived from the NICE and PECARN guidelines. Abbreviations: ci-TBI: clinically important traumatic brain injury.

1: Fall > 0.9 m if age < 2 years or > 1.5 m if age ≥ 2 years; motor vehicle accident with ejection, fatality, rollover; bike or pedestrian versus vehicle without helmet; struck by high impact object.

2: headache, vomiting ; agitation, somnolence, slow response, repetitive questioning.

A. Under 2 years

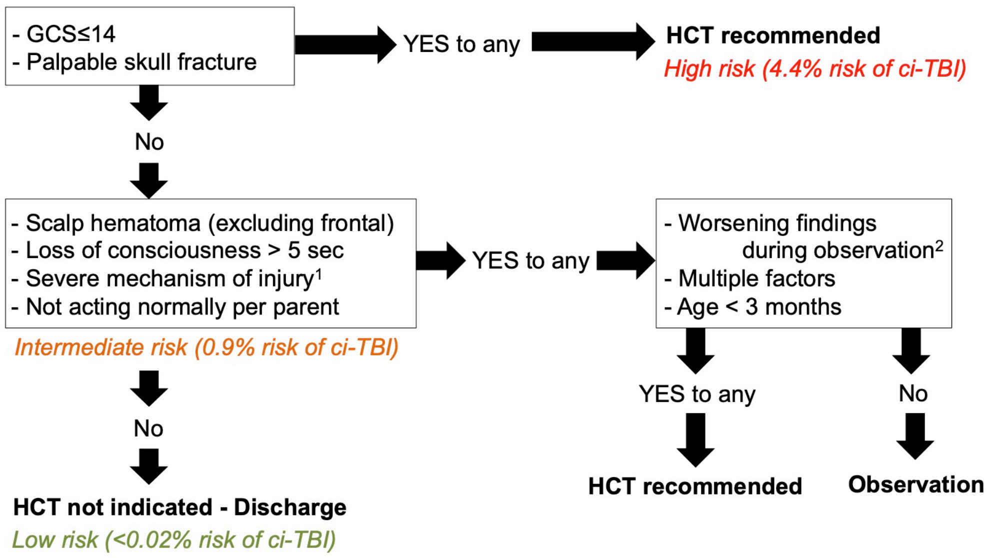

B. Above 2 years

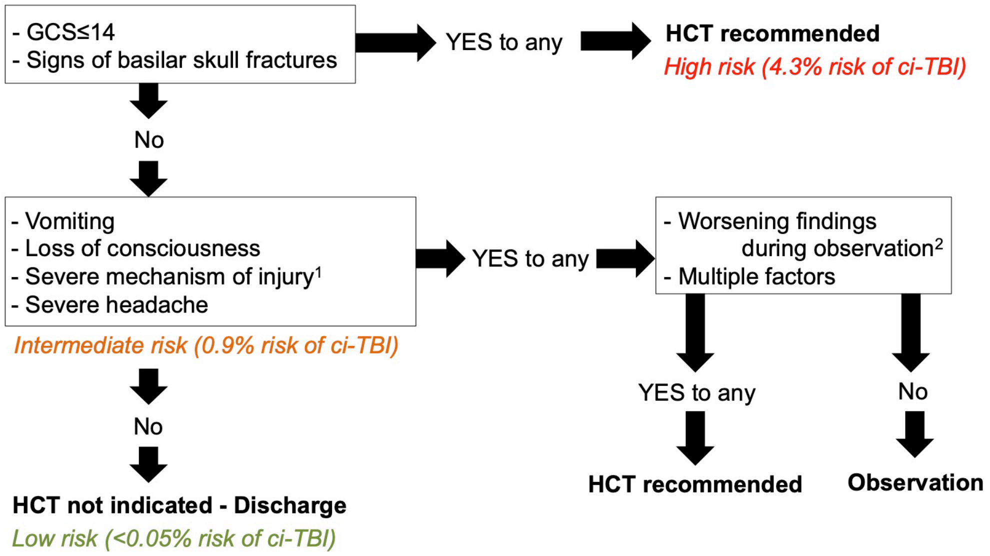

© 2023 Roche S et al. *JAMA Network Open.*

eTable 2. Associations Between Main Findings on Head Computed Tomography and Patients' Age

Characteristics	Age groups 0 - 2 years (n=659) No. (%)	2 - 6 years (n=927) No. (%)	6 - 12 years (n=1,137) No. (%)	12 - 18 years (n=2,423) No., (%)	Adjusted P_{CA}	Adjusted P_{X2}
**Intracranial hemorrhage**	84 (12.7)	61 (6.6)	73 (6.4)	88 (3.6)	<.001	<.001
**Extradural hematoma**					<.001	<.001
No	622 (94.4)	900 (97.1)	1,108 (97.4)	2,390 (98.6)		
Yes	37 (5.6)	27 (2.9)	29 (2.6)	33 (1.4)		
**Subdural hematoma**					<.001***	<.001
No	619 (93.9)	900 (97.1)	1,115 (98.1)	2,383 (98.3)		
Yes	40 (6.1)	27 (2.9)	22 (1.9)	40 (1.7)		
**Subarachnoid hemorrhage**					.007	.02
No	641 (97.3)	912 (98.4)	1,114 (98)	2,397 (98.9)		
Yes	18 (2.7)	15 (1.6)	23 (2)	26 (1.1)		
**Intraparenchymal hemorrhage**					.30	0.72
No	658 (99.8)	924 (99.7)	1,133 (99.6)	2,412 (99.5)		
Yes	1 (0.2)	3 (0.3)	4 (0.4)	11 (0.5)		
**Petechiae**					.32	0.50
No	653 (99.1)	918 (99)	1,118 (98.3)	2,391 (98.7)		
Yes	6 (0.9)	9 (1)	19 (1.7)	32 (1.3)		
**Fractures**					<.001	<.001
No	529 (80.3)	801 (86.4)	1,002 (88.1)	2,140 (88.3)		
Yes	130 (19.7)	126 (13.6)	135 (11.9)	283 (11.7)		
**Facial bone fracture**					<.001	<.001
No	655 (99.4)	885 (95.5)	1,071 (94.2)	2,206 (91)		
Yes	4 (0.6)	42 (4.5)	66 (5.8)	217 (9)		
**Skull base fracture**					.14	.21
No	642 (97.4)	899 (97)	1,103 (97)	2,376 (98.1)		
Yes	17 (2.6)	28 (3)	34 (3)	47 (1.9)		
**Upper cervical spine fracture**					.31	.72
No	659 (100)	926 (99.9)	1,136 (99.9)	2,419 (99.8)		
Yes	0 (0)	1 (0.1)	1 (0.1)	4 (0.2)		
**Skull vault fracture**					<.001	<.001
No	543 (82.4)	844 (91)	1,076 (94.6)	2,361 (97.4)		
Yes	116 (17.6)	83 (9)	61 (5.4)	62 (2.6)		
**Suture disjunction**					0.06	.31
No	653 (99.1)	921 (99.4)	1,132 (99.6)	2,415 (99.7)		
Yes	6 (0.9)	6 (0.6)	5 (0.4)	8 (0.3)		
**Extracranial hematoma**					<.001	<.001
No	577 (87.6)	871 (94)	1,086 (95.5)	2,342 (96.7)		
Yes	82 (12.4)	56 (6)	51 (4.5)	81 (3.3)		

© 2023 Roche S et al. *JAMA Network Open.*

NOTE.- P_{CA} corresponds to the P-value of the Cochrane-Armitage test and P_{X2} to the classical Chi-square P-value. P-values were adjusted for multiple comparisons (herein 13 tests) using the Benjamini-Hochberg procedure. Abbreviation: No.: number.

© 2023 Roche S et al. *JAMA Network Open.*

eTable 3. Associations Between Main Findings on Head Computed Tomography and Patients' Sex

Characteristics	Sex		
	Girls (n=1,899) No. (%)	Boys (n=3,245) No. (%)	Adjusted P_{X2}
**Intracranial hemorrhage**			.30
No	1,798 (94.7)	3,040 (93.7)	
Yes	101 (5.3)	205 (6.3)	
**Extradural hematoma**			.56
No	1,857 (97.8)	3,161 (97.4)	
Yes	42 (2.2)	84 (2.6)	
**Subdural hematoma**			.13
No	1,862 (98.1)	3,153 (97.2)	
Yes	37 (1.9)	92 (2.8)	
**Subarachnoid hemorrhage**			.96
No	1,868 (98.4)	3,194 (98.4)	
Yes	31 (1.6)	51 (1.6)	
**Intraparenchymal hemorrhage**			.33
No	1,895 (99.8)	3,230 (99.5)	
Yes	4 (0.2)	15 (0.5)	
**Petechiae**			.33
No	1,880 (99)	3,198 (98.6)	
Yes	19/1,899 (1)	47 (1.4)	
**Fractures**			<.001
No	1,704 (89.7)	2,766 (85.2)	
Yes	195 (10.3)	479 (14.8)	
**Facial bone fracture**			<.001
No	1,812 (95.4)	3,003 (92.5)	
Yes	87 (4.6)	242 (7.5)	
**Skull base fracture**			.04
No	1,867 (98.3)	3,151 (97.1)	
Yes	32/1,899 (1.7)	94 (2.9)	
**Upper cervical spine fracture**			.59
No	1,898 (99.9)	3,240 (99.8)	
Yes	1 (0.1)	5/3,245 (0.2)	
**Skull vault fracture**			.03
No	1,802 (94.9)	3,020 (93.1)	
Yes	97 (5.1)	225 (6.9)	
**Suture disjunction**			.56
No	1,892 (99.6)	3,227 (99.4)	
Yes	7 (0.4)	18 (0.6)	
**Extracranial hematoma**			.048
No	1,818 (95.7)	3,056 (94.2)	
Yes	81 (4.3)	189 (5.8)	

NOTE.- P_{X2} to the classical Chi-square P-value since sex was not an ordinal variable. P-values were adjusted for multiple comparisons (herein 13 tests) using the Benjamini-Hochberg procedure. Abbreviation: No.: number.
Please note that the sex of two children was unknown.

© 2023 Roche S et al. *JAMA Network Open.*

eTable 4. Associations Between Main Findings on Head Computed Tomography and Patients' Glasgow Coma Scale

Characteristics	Glasgow score (GCS) GCS = 15 (n=3,211) No. (%)	13 ≤ GCS ≤ 14 (n=301) No. (%)	9 ≤ GCS ≤ 12 (n=91) No. (%)	3 ≤ GCS ≤8 (n=40) No. (%)	Adjusted P_{CA}	Adjusted P_{X2}
**Intracranial hemorrhage**					<.001	.001
No	3,027 (94.3)	273 (90.7)	80 (87.9)	33 (82.5)		
Yes	184 (5.7)	28 (9.3)	11 (12.1)	7 (17.5)		
**Extradural hematoma**					.045	.20
No	3,137 (97.7)	289 (96)	87 (95.6)	38 (95)		
Yes	74 (2.3)	12 (4)	4 (4.4)	2 (5)		
**Subdural hematoma**					.006	.01
No	3,131 (97.5)	284 (94.4)	86 (94.5)	38 (95)		
Yes	80 (2.5)	17 (5.6)	5 (5.5)	2 (5)		
**Subarachnoid hemorrhage**					<.001	.002
No	3,165 (98.6)	294 (97.7)	86 (94.5)	37 (92.5)		
Yes	46 (1.4)	7 (2.3)	5 (5.5)	3 (7.5)		
**Intraparenchymal hemorrhage**					.67	.33
No	3,196 (99.5)	300 (99.7)	91 (100)	39 (97.5)		
Yes	15 (0.5)	1 (0.3)	0 (0)	1 (2.5)		
**Petechiae**					<.001	<.001
No	3,176 (98.9)	297 (98.7)	90 (98.9)	35 (87.5)		
Yes	35 (1.1)	4 (1.3)	1 (1.1)	5 (12.5)		
**Fractures**					.06	.004
No	2,806 (87.4)	239 (79.4)	77 (84.6)	36 (90)		
Yes	405 (12.6)	62 (20.6)	14 (15.4)	4 (10)		
**Facial bone fracture**					.36	.06
No	3,017 (94)	270 (89.7)	86 (94.5)	38 (95)		
Yes	194 (6)	31 (10.3)	5 (5.5)	2 (5)		
**Skull base fracture**					.01	.02
No	3,145 (97.9)	287 (95.3)	86 (94.5)	39 (97.5)		
Yes	66 (2.1)	14 (4.7)	5 (5.5)	1 (2.5)		
**Upper cervical spine fracture**					>.99	.86
No	3,206 (99.8)	300 (99.7)	91 (100)	40 (100)		
Yes	5 (0.2)	1 (0.3)	0 (0)	0 (0)		
**Skull vault fracture**					.045	.10
No	3,021 (94.1)	273 (90.7)	82 (90.1)	37 (92.5)		
Yes	190 (5.9)	28 (9.3)	9 (9.9)	3 (7.5)		
**Suture disjunction**					.46	.53
No	3,197 (99.6)	298 (99)	90 (98.9)	40 (100)		
Yes	14 (0.4)	3 (1)	1 (1.1)	0 (0)		
**Extracranial hematoma**					.83	.71
No	3,039 (94.6)	280 (93)	87 (95.6)	38 (95)		
Yes	172 (5.4)	21 (7)	4 (4.4)	2 (5)		

© 2023 Roche S et al. *JAMA Network Open.*

NOTE.- P_{CA} corresponds to the P-value of the Cochrane-Armitage test and P_{X2} to the classical Chi-square P-value. P-values were adjusted for multiple comparisons (herein 13 tests) using the Benjamini-Hochberg procedure. Please note that the GCS was not available for 1503 patients.

© 2023 Roche S et al. *JAMA Network Open.*

eFigure 2. HCT for 21-month-old Patient

A 21-month-old patient presenting with a brain trauma without loss of consciousness in the context of a fall from a chair (80 cm heighth). Clinically, the patient displayed an occipital subcutaneous hematoma and jet vomiting. The GCS is 15. (**A**) Soft tissue hematoma (blue arrow) on coronal view of the head computed tomography (HCT) – brain window. (**B**) Occipital linear skull fracture (pink arrow) with pneumencephalia (green arrow) on axial view – bone window. (**C**) Left peri-cerebellum extra dural hematoma (red arrow), on axial view – brain window. (**D**) Occipital linear skull fracture with reconstruction 3D volume rendering mode (violet arrows)

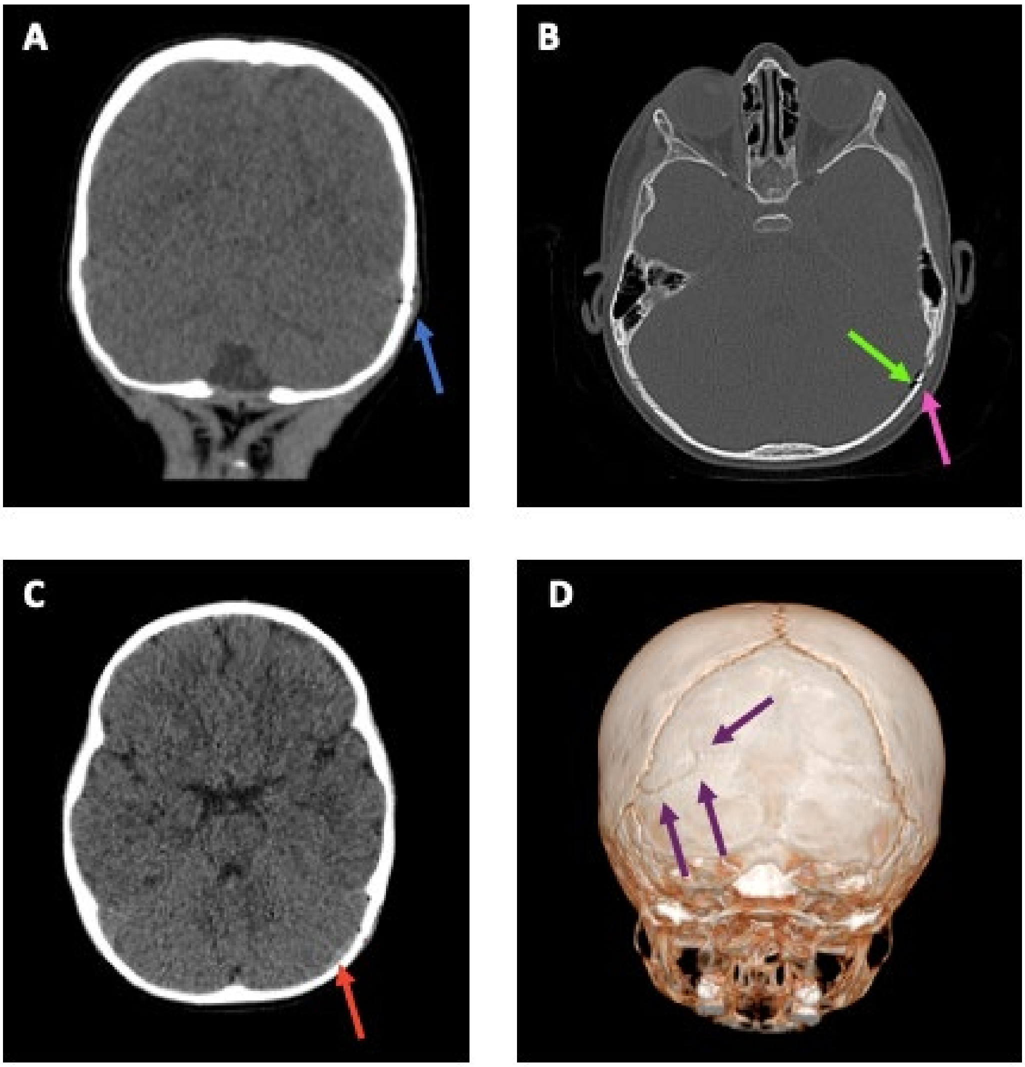

© 2023 Roche S et al. *JAMA Network Open.*

eFigure 3. HCT for 15-year-old Patient

A 15-years-old patient presenting at the emergency department after an aggression. He showed a left frontal wound with clinical suspicion of embarrure and fracture of the facial mass involving the left orbit. The GCS was 15 without neurological deficit. (**A**) Soft tissue hematoma (blue arrow) on axial head computed tomography (HCT) – brain window. (**B**) Left frontal embarrure fracture (green arrow) associated with a dental avulsion fracture (42) (pink arrow) on coronal view - bone window. (**C**) Petechiae (red arrow), subarachnoid hematoma (orange arrow) and sub-dural hematoma (yellow arrow) on axial view - brain window. (**D**) Linear fracture of right C3 transverse foramen (pink arrows) on axial view - bone window.

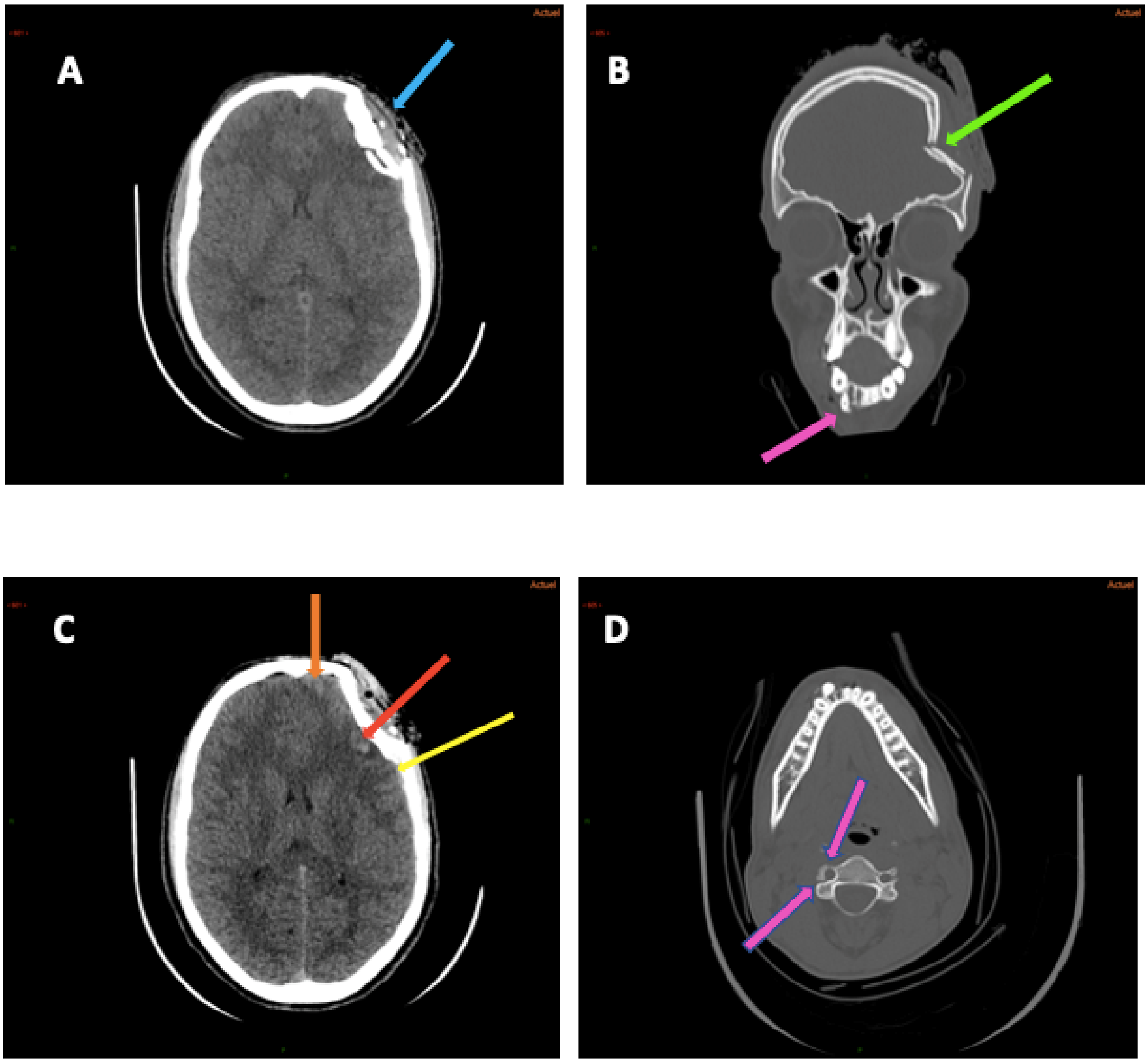

© 2023 Roche S et al. *JAMA Network Open.*

eTable 5. Characteristics of the 600 HCT Requests Randomly Sampled to Investigate the Implementation of the PECARN Rules

Characteristics	Subcohort for indication (n=589) No. (%)
**Patients' characteristics**	
**Age, median (Q1-Q3), y**	11.1 (5 - 15.8)
**Age categories**	
0 - 1	70 (11.9)
2 - 5	97 (16.5)
6 - 11	143 (24.3)
12 - 17	279 (47.4)
**Sex**	
Girls	228 (38.7)
Boys	361 (61.3)
**Glasgow coma scale**	
15	548 (93)
13 - 14	31 (5.3)
9 - 12	8 (1.4)
3 - 8	2 (0.3)
**Examination characteristics**	
**Emergency level**	
Extreme emergency (level 1)	19 (3.2)
Usual emergency (level 2)	536 (91)
Organizational emergency (level 3)	34 (5.8)
**Contrast medium injection**	
Yes	8 (1.4)
No	581 (98.6)
**Other acquisition(s) performed during CT examination**	
Abdomen pelvic	1 (0.2)
Thorax abdomen pelvic	1 (0.2)
Chest	2 (0.3)
**Radiological findings**	
**Pathological findings**	
No	478 (81.2)
Yes, related to the trauma	101 (17.1)
Yes, but fortuitous, unrelated to trauma	10 (1.7)
**Intra-cranial hemorrhage (ICH)**	32 (5.4)
**ICH types**	
Subdural hematoma	12 (2)
Extradural hematoma	15 (2.5)
Subarachnoid hemorrhage	9 (1.5)
Petechiae	3 (0.5)
Intra-parenchymal hemorrhage	1 (0.2)
Intraventricular hemorrhage	0 (0)
**No. of distinct ICH types**	
1	26 (4.4)
2	4 (0.7)
3	2 (0.3)
4	0 (0)
**Fracture**	81 (13.8)
**Fracture types**	
Facial bone	43 (7.3)
Skull vault	35 (5.9)
Skull base	14 (2.4)
Suture disjonction	3 (0.5)
Upper cervical spine	0 (0)

© 2023 Roche S et al. *JAMA Network Open.*

Characteristics	Subcohort for indication (n=589) No. (%)
**No. of distinct fracture types**	
1	69 (11.7)
2	10 (1.7)
3	2 (0.3)
4	0 (0)
**Other intracranial traumatic lesions**	
Pneumencephalia	9 (1.5)
Subfalcine herniation	1 (0.2)
Temporal herniation	1 (0.2)
Central herniation	1 (0.2)
Cerebral edema	2 (0.3)
hydrocephalia	0 (0)
Diffuse axonal lesions	1 (0.2)
**Extracranial hemorrhage**	32 (5.4)

NOTE.- Abbreviations: ICH: intra-cranial hemorrhage; No.: number; Q1-Q3: interquartile range. Data are number of patients with percentage in parentheses, except for numeric variables.

Please note that 11 HCT requests out of the 600 sampled ones were not analyzable because of incomprehensible or missing indications.

eTable 6. Patterns of Traumatic Brain Injuries on HCT Diagnosed in the 3 Groups Depending on the Theoretical Management up to PECARN Algorithm as Used in France

Characteristics	Theorical management according to guidelines		
	Discharge n=226 No. (%)	Monitoring at hospital n=170 No. (%)	HCT recommended n=193 No. (%)
**Intracranial hemorrhage (ICH)**	11 (4.9)	8 (4.7)	13 (6.7)
**Type of ICH**			
Extradural hematoma	6 (2.7)	5 (2.9)	4 (2.1)
Subdural hematoma	2 (0.9)	3 (1.8)	7 (3.6)
Subarachnoid hemorrhage	2 (0.9)	2 (1.2)	5 (2.6)
Intraparenchymal hematoma	1 (0.4)	0 (0)	0 (0)
Petechiae	3 (1.3)	0 (0)	0 (0)
**Fracture**	29 (12.8)	19 (11.2)	33 (17.1)
**Type of fracture**			
Facial bone	19 (8.4)	10 (5.9)	14 (7.3)
Skull base	4 (1.8)	2 (1.2)	8 (4.1)
Skull vault	12 (5.3)	7 (4.1)	16 (8.3)
Suture disjunction	1 (0.4)	2 (1.2)	0 (0)
**Other significant lesions**			
Diffuse cerebral edema	0 (0)	0 (0)	2 (1)
Diffuse axonal lesion	0 (0)	0 (0)	1 (0.5)
Subfalcine herniation	0 (0)	0 (0)	1 (0.5)
Temporal herniation	0 (0)	0 (0)	1 (0.5)
Central herniation	0 (0)	1 (0.6)	0 (0)
pneumocephalus	2 (0.9)	1 (0.6)	6 (3.1)

© 2023 Roche S et al. *JAMA Network Open.*
