# Supplementary material for: Risk Factors Associated with Traumatic Brain Injury and Implementation of Guidelines for Requesting Computed Tomography After Head Trauma Among Children in France
Source: JAMA Netw Open. 2023 May 2;6(5):e2311092. doi: 10.1001/jamanetworkopen.2023.11092 (PMC10155067; doi:10.1001/jamanetworkopen.2023.11092)
Supplement: Supplement 2. — Data Sharing Statement [file jamanetwopen-e2311092-s002.pdf]

Data Sharing Statement

Roche. Risk Factors Associated with Traumatic Brain Injury and Implementation of Guidelines for Requesting Computed Tomography After Head Trauma Among Children in France. *JAMA Netw Open*. Published May 02, 2023. doi:10.1001/jamanetworkopen.2023.11092

Data
Data available: Yes
Data types: Deidentified participant data
How to access data: Data generated and analyzed during the study are available from the corresponding author upon request : g.gorincour@imadis.fr
When available: With publication

Supporting Documents
Document types: None

Additional Information
Who can access the data: researchers whose proposed use of the data has been approved
Types of analyses: for any purpose
Mechanisms of data availability: after approval of a proposal. Depending on needs of researchers: collaboration, support and agreement could be implemented
